# Investigation of viewing procedures for interpretation of breast tomosynthesis image volumes: a detection-task study with eye tracking

**DOI:** 10.1007/s00330-012-2675-z

**Published:** 2012-10-20

**Authors:** Pontus Timberg, Kristina Lång, Marcus Nyström, Kenneth Holmqvist, Philippe Wagner, Daniel Förnvik, Anders Tingberg, Sophia Zackrisson

**Affiliations:** 1 Diagnostic Radiology, Lund University, Skåne University Hospital, 205 02 Malmö, Sweden; 2 Diagnostic Centre of Imaging and Functional Medicine, Lund University, Skåne University Hospital, 205 02 Malmö, Sweden; 3 The Humanities Laboratory, Lund University, Box 201, 221 00 Lund, Sweden; 4 National Competence Center for Musculoskeletal Disorders, Department of Orthopedics, Lund University, 221 85 Lund, Sweden; 5 Medical Radiation Physics, Lund University, Skåne University Hospital, 205 02 Malmö, Sweden; 6 Diagnostic Centre of Imaging and Functional Medicine, Lund University, Skåne University Hospital, 205 02 Malmö, Sweden

**Keywords:** Breast tomosynthesis, Image volumes, Reading frames, Perception, Eye tracking

## Abstract

**Objectives:**

To evaluate the efficiency of different methods of reading breast tomosynthesis (BT) image volumes.

**Methods:**

All viewing procedures consisted of free scroll volume browsing and three were combined with initial cine loops at three different frame rates (9, 14 and 25 fps). The presentation modes consisted of vertically and horizontally orientated BT image volumes. Fifty-five normal BT image volumes in mediolateral oblique view were collected. In these, simulated lesions were inserted, creating four unique image sets, one for each viewing procedure. Four observers interpreted the cases in a free-response task. Time efficiency, visual attention and search were investigated using eye tracking.

**Results:**

Horizontally orientated BT image volumes were read faster than vertically when using free scroll browsing only and when combined with fast cine loop. Cine loops at slow frame rates were ruled out as inefficient.

**Conclusions:**

In general, horizontally oriented BT image volumes were read more efficiently. All viewing procedures except for slow frame rates were promising when assuming equivalent detection performance.

***Key Points*:**

• *Breast tomosynthesis is increasingly used for breast cancer detection*

• *There is a benefit in reading breast tomosynthesis image volumes presented horizontally*

• *Align image content to visual field, especially for dynamic 3D images*

• *Reading at slow frame rates was considered inefficient*

## Introduction

Mammography is the established method in breast cancer screening and has potential to reduce breast cancer mortality [1–3]. Still, it has a false-negative rate of 15–30 % [4–6]. This is mainly due to difficulties in detecting abnormalities in breasts with dense and superimposed tissue. These problems can be reduced with a three-dimensional (3D) technique called breast tomosynthesis (BT) [7, 8]. With this technique the X-ray tube moves in an arc over a limited angular range of the breast during exposure, acquiring a number of projection images. These images are used to reconstruct a 3D BT image volume, from which thin slices can be viewed.

Breast tomosynthesis has been shown to increase sensitivity of breast cancer detection compared with digital mammography (DM) in selected materials [9, 10]. Whether BT can be used in screening is under investigation. To our knowledge, there are two large, ongoing clinical screening trials in Scandinavia aimed at answering that question (ClinicalTrials.gov id: NCT01091545; NCT01248546). One major problem that arises with BT is the extensive set of data generated compared with DM. Reading time is considered longer for BT than for DM [11]. If BT is to be used in screening, the need for optimising reading conditions becomes evident.

BT image volumes can be viewed using free scroll volume browsing (FS) or in a cine loop. Currently, the standard procedure is FS, as it is necessary when confirming findings. However, in clinical practice, observers often utilises a cine loop to get an overview before carrying on with a more scrutinising search. How different frame rates are related to accuracy in detecting lesions has been investigated in CT [12, 13]. These studies indicate that a higher frame rate reduces accuracy, when analysing frame rates from 0.5 to 21 frames per second (fps). How different frame rates are related to diagnostic accuracy in BT image volumes and if there are any benefits of showing initial cine loops have not yet been investigated.

BT image volumes could be displayed on the monitor in either vertical or horizontal orientation. Peripheral vision guides visual search [14–16] and it has been confirmed in visual search tasks that the perceptual span is larger for horizontal than for vertical searches [17, 18]. Traditionally, wide screen monitors are orientated vertically in radiology reading settings. This may influence efficiency and accuracy in interpreting an image, but has not yet been investigated. Theoretically, viewing horizontally orientated presentations of BT image volumes makes reading more efficient because the extension of the visual field is better aligned with the viewing area of the monitor. Such alignment seems particularly useful when viewing dynamically presented BT image volumes where abnormalities appear abruptly in the peripheral part of the visual field (similar effects have been found in industrial inspection [19]). Such abrupt onsets are known to capture attention [20]. This might be reflected in faster total analysis time and shorter entry time (time from case onset until the region of interest [ROI] is visually localised).

When estimating how BT image volumes are perceived, eye tracking can be utilised. Eye trackers consist of an infrared camera that films the eye, and software that calculates the gaze position using image processing and geometrical calculations. The recorded imaging path can be visualised and analysed statistically [21]. This can provide an understanding of how we perceive and analyse visual input, e.g. radiological images [22–31].

The purpose of this study was to evaluate the efficiency of several BT image volume readings using an experimental setup. We evaluated these in terms of lesion detection performance, time efficiency, visual attention and search using Jack-knife Alternative Free-response Receiver Operating Characteristics (JAFROC) and eye tracking.

## Materials and methods

### Image collection and preparation

Fifty-five normal BT cases in mediolateral oblique view were selected. These cases were verified by an expert radiologist panel (minimum follow-up of 4 years). The mean age of the women was 58 years (range 33–75 years) with a mean breast thickness of 45 mm (range 19–69 mm). The examinations were acquired with a Siemens Mammomat Novation BT prototype (Siemens Healthcare, Erlangen, Germany), using a tungsten/rhodium anode/filter combination. The exposure was set at an average glandular dose of approximately 1.6 mGy for a 50 mm standard breast. The patient selection criteria have been described in detail earlier [9]. The study protocol was approved by the Regional Ethics Review Board at Lund University (Dnr 159/2006) and the local Radiation Safety Committee at Skåne University Hospital, Malmö.

**Fig. 1:**
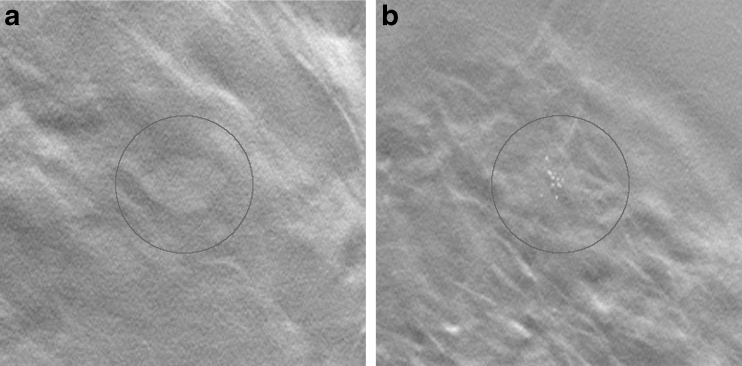
Reconstructed slices of simulated lesions presented within the circle: (**a**) mass and (**b**) microcalcification cluster

In these BT image volumes, simulated 3D distributed lesions (20 masses and 20 clusters of microcalcifications) were randomly inserted (Fig. 1), creating four unique image sets (one for each viewing procedure) and a total of 160 unique lesion locations, as described below. Possible locations were enclosed by the skin line and the pectoralis muscle. To give a more realistic appearance, the microcalcification clusters were manually moved to the closest dense area of the random insertion point.

The lesions were inserted according to previously described methods [32, 33] with some new modifications: scatter compensation and search factor (SF). The microcalcification clusters consisted of 15 individual calcifications with an average size of 0.2 mm. The positions of the calcifications were normally distributed and the cluster was randomly inserted into dense regions of the breast volume. The irregularly shaped masses had an average size of 8 mm and were inserted randomly. The primary contrasts (Cp) of the lesions were derived from a 3D four-alternative forced-choice task [32, 34] for clusters and 8-mm masses (Cp_mass_ = 0.034 and Cp_calcs_ = 0.041 at a detectability index of 2.5). The contrasts (C) were then calculated by correcting for scatter, depending on the breast thickness, independent of beam quality and breast density. These contrasts had to be multiplied with an arbitrary SF to make it possible to detect the lesions when searching through entire BT volumes. An estimate was derived from a free search model [35] in breast images indicating that about five times more contrast is needed compared with a two-alternative forced-choice task for 8-mm lesions. However, when conducting our particular detection task, this factor was increased to seven times for masses and five times for microcalcification clusters to end up with a JAFROC FOM of around 0.7–0.9. The contrast was adjusted as follows:

$$ \mathrm{C}=\mathrm{Cp}/\left( {1+\mathrm{SPR}} \right) \times \mathrm{SF}, $$

where the scatter-to-primary ratio (SPR) for a given breast thickness was estimated as presented by Boone et al. [36] and Wu et al. [37]. The SPR was 0.46 for a 4 cm breast with 50 % glandularity at 27 kVp at projection angle of 0°. Finally, the five outermost slices of all image sets were removed as suggested by local radiologist experts, because they do not usually provide any data of interest. All image sets were reconstructed using filtered backprojection, with a voxel size of 0.085 mm × 0.085 mm × 1 mm. Two Hanning filters were employed [38]; a spectral filter with a cut-off frequency of 1.5 × Nyquist frequency (f_Ny_) and a slice thickness filter of 0.7 × f_Ny_.

### Observer study setup

The BT image volume presentation modes consisted of vertically orientated images and horizontally orientated images. Each presentation mode was analysed with four different viewing procedures consisting of:

1. Free scroll volume browsing (FS)
2. Viewing of an initial cine loop at fast frame rate (25 fps), followed by additional FS
3. Initial cine loop at medium frame rate (14 fps), followed by additional FS
4. Initial cine loop at slow frame rate (9 fps), followed by additional FS

The frame rates were set by the maximum frame rate supported by the capacity of the designated workstation and by the minimum frame rate recommended by a senior radiologist (not participating in the study). For each viewing procedure a unique image set was created (all image sets were displayed as a left mediolateral oblique view of the breast). This generated a total of eight trials with 55 BT cases in each, which were presented in random order per observer and session.

One general radiologist, one 2nd-year resident) and two medical physicists participated in all trials. There were no dedicated breast radiologists participating in the study for practical reasons and it was not required for the experimental setup because known and well-defined simulated lesions were used. Their task was to detect the lesions in the BT image volumes utilising a free-response task. They were told to ignore other findings in the breast, such as lymph nodes in the axilla and adjacent to the pectoral muscle and benign calcifications. For practical reasons, they were informed that there was at the most one lesion per case. Suspicious findings were marked and rated, using a five-level confidence scale: definitively not a lesion, probably not a lesion, probably a lesion, likely to be a lesion, definitively a lesion. In the FS procedure, suspicious findings were marked and rated, whereas in the cine loop mode a two-step evaluation was used:

1. Mark suspicious findings found in the initial cine loop, by using sequential FS, and assign confidence rating
2. Mark additional findings found during the sequential FS procedure only

Before each condition a training session was provided, consisting of about ten cases.

### User interface and eye tracking

**Fig. 2:**
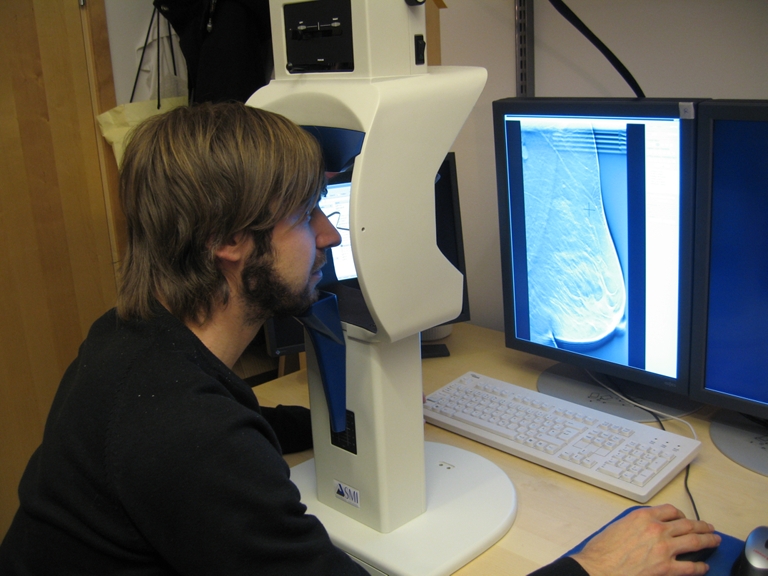
Eye tracking setup in action

**Fig. 3:**
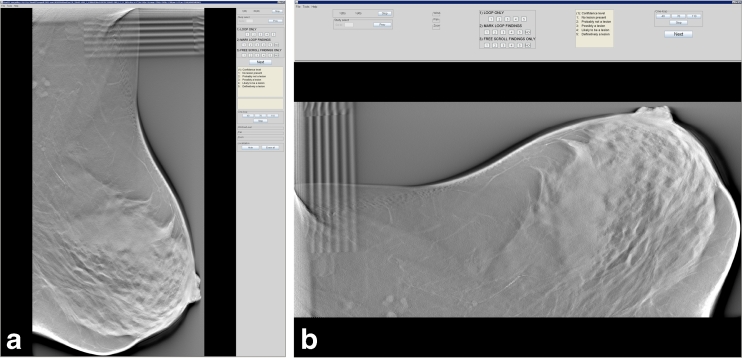
**a** The ViewDEX user interface with the task panel on the right hand side. This breast tomosynthesis (BT) case is presented in a vertical position. **b** A BT volume slice shown in a horizontal position, now with the task panel at the top. Reprinted from Lång K. et al. Proc. SPIE 7966:796606.1-12, 2011

Eye movement data were recorded with an SMI HiSpeed 240 tower mounted eye tracker (SensoMotoric Instruments, Teltow, Germany) (Fig. 2). The recording computer ran the SMI iView X 2.2 software in pupil–corneal reflection mode and otherwise default settings (www.smivision.de). A modified version of the ViewDEX [39, 40] user interface was developed, synchronised with the eye tracking system and set up to send eye tracking commands (Fig. 3). The eye tracker had a sampling frequency of 240 Hz and measured accuracy of <0.3° of visual angle. The eye tracking device was calibrated at the beginning and halfway through the session using a 13-point calibration method in iView X. From the recorded eye-movement data and known positions of lesions we extracted measures of visual attention and search. Because all presentation modes include dynamic stimuli, which could elicit smooth pursuit eye movement (slow motion of the eye as it follows something moving), we analysed the ‘raw’ eye movement data directly. Currently, there are no robust methods of extracting fixations from data containing smooth pursuit eye movements [21].

All images were displayed proportionally scaled into a region of 1,698 by 2,000 pixels (0.165 mm × 0.165 mm) on a DICOM calibrated 5-megapixel flat panel EIZO SMD 21510 monitor in a room with an ambient light level around 3 lux. It was positioned at a distance of 62 cm from the observer. The observers could not alter the window/level setting during the study.

### Analysis of detection performance data

**Fig. 4:**
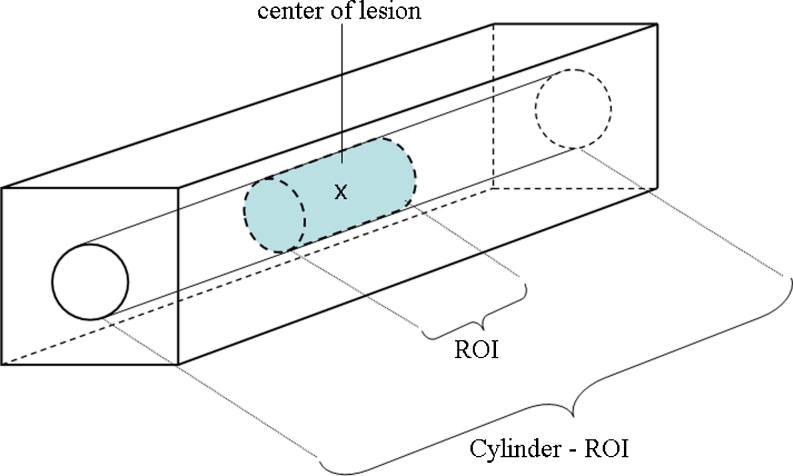
Schematic illustration of the BT image volumes showing the position of the lesion, the ROI and the c-ROI. Reprinted from Lång K. et al. Proc. SPIE 7966:796602.1-11, 2011

An ROI was defined as 1.5° from the lesion edge (lesion size was approximated as 1°), giving a total radius of 2°. Three additional slices above and below were included, forming a cylinder. Marks made inside the ROI were considered a lesion localisation, elsewhere as a non-lesion localisation. An extension of this cylinder throughout the breast in the depth direction was named the cylinder ROI (c-ROI). In the cine loop procedure, the visual attention of an observer can be caught to a c-ROI awaiting the appearance of a lesion (Fig. 4).

The detection performance was analysed using JAFROC [41]. The observer-averaged JAFROC figure of merit (FOM) and its confidence intervals CI_95_ were calculated together with the lesion localisation fraction (LLF) relative to the total number of lesions, for both masses and microcalcification clusters, and non-lesion localisation fraction (NLF), relative to the number of images. The difference between the rating of the initial cine loop and the rating of additional FS in the same session was compared to test for statistical significance. In this study, we only compared paired data (e.g. data for each of the presentation modes and within the same session), because a different linear analysis model from JAFROC is required for unpaired data. Based on the limited data set, we did not expect to find any statistically significant differences in FOM between the sessions and decided only to provide brief results.

### Analysis of data related to time efficiency and visual attention and search

Differences in time efficiency for the viewing procedures and presentation modes were measured using *total analysis time*. It was defined as the observer’s time spent per case until proceeding to next case [26]. The time spent in the menu was excluded. The median total analysis time and the time spent in cine loop were measured.

*Total dwell time* was the total time spent by the observers, including revisits, in the ROI and in the c-ROI, per abnormality. It reflects the effect of the viewing procedure on visual attention for more or less conspicuous lesions [21]. In order to compare all frame rates, a relative measure of the total dwell time, normalised to the total time spent in the cine loop (independent of breast thickness and frame rate) was used. Pooled data from both presentation modes were analysed as the data sets provided similar results (no statistically significant differences) when analysed separately. We hypothesised that a higher proportion of dwell time in the c-ROI/ROI would lead to better detection performance.

*Entry time* was measured as the time from case onset until the ROI was visually localised [22]. To prevent localisations triggered by saccades and very short fixations, only dwells inside the ROI longer than 100 ms were counted (Table 2). We hypothesised that earlier localisation of the target indicates faster detection, and hence shorter total analysis time.

*Transition length* was defined as the distance between the gaze position when the centre of the lesion was onset and the ROI border. Only transitions longer than 3° and completed within 500 ms were included, likely to result from the lesion onset. The transition lengths were analysed for all observers, abnormal cases and for different image presentations in the cine loop viewing modes (Fig. 9). Longer transitions are indicative of utilising more of the peripheral visual field as guidance for foveal search [18, 42]. We expect longer transitions in horizontal presentations and that masses generate stronger transient onsets in dynamic presentations whereas microcalcification clusters requires a systematic search strategy using shorter transitions.

The objective of the statistical analysis was to test differences in outcomes of total analysis time, entry time, transition lengths, total dwell time (in ROI and c-ROI) with respect to viewing procedures and presentation modes. Owing to the different nature of the data of these outcomes, different methods of statistical analysis were applied to each dataset. All outcomes except dwell times were log-transformed. Because of the correlation structure of the time and entry time data, due to multiple observers studying the same cases, a customised two-level linear random effects model (made in R version 2.13.0, www.R-project.org) was used to analyse these two outcomes. One random effect was added to account for correlation between responses from the same observer studying different cases and one to account for correlation between different observers studying the same case. For all analyses, vertical FS (and vertical fast frame rate mode for some conditions) was used as reference level for comparison with other modes. Estimates from all analyses can be interpreted in the same manner on a relative scale. For instance, if the estimate for the medium frame rate in horizontal presentation mode in the analysis of time were 1.5, this would mean that this mode takes 50 % more time than the fast vertical presentation mode.

## Results

### Detection performance

**Fig. 5:**
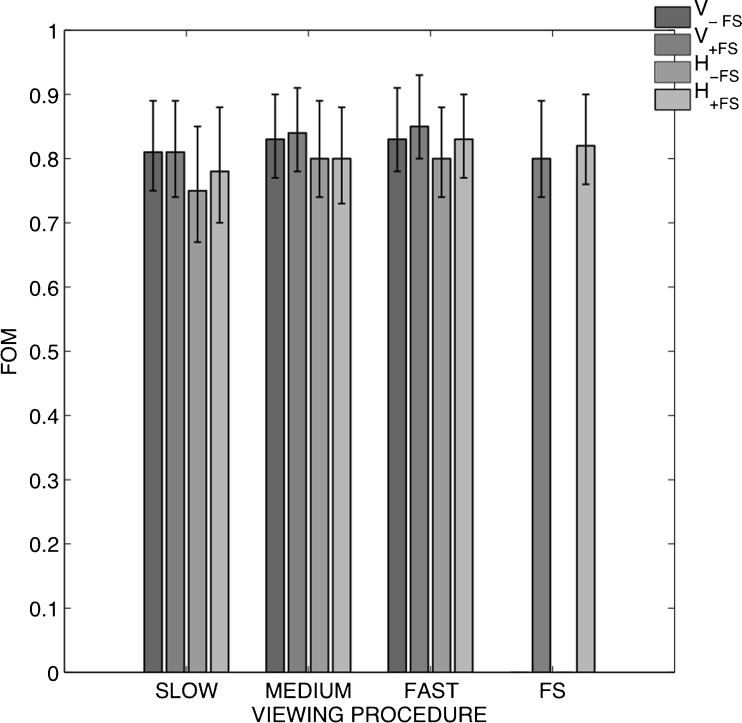
Results from JAFROC analysis. JAFROC FOM and CI_95_ for all conditions. The *bars* are gathered per viewing procedure, including both detection in the initial cine loop (−FS) and combined with free scroll browsing (+FS). The *two rightmost bars* indicate the free scroll viewing procedures

The observed differences in detection performance (FOM of 0.75–0.86) were not statistically significant between any reading condition (Fig. 5). The LLFs and NLFs are presented in Table 1. Compared with vertical image presentation, horizontal image presentation had overall slightly lower FOMs mainly because of an overall tendency towards increased NLF. Most masses were found at fast frame rates, whereas microcalcification clusters in general were better discerned at medium frame rates.

**Table 1:** Lesion localisation fraction (*LLF*), non-lesion localisation fraction (*NLF*) in cine loops and combined with free scroll browsing. The proportions of located microcalcification clusters and masses (microcalcification clusters LLF/masses LLF) are presented next to the LLFs

Presentation mode	Viewing procedure	Cine loop LLF	Cine loop NLF	Cine loop + free scroll LLF	Cine loop + free scroll NLF
Vertically	Slow	0.66(0.68/0.64)	0.07	0.69(0.74/0.65)	0.10
	Medium	0.69(0.69/0.70)	0.06	0.74(0.78/0.70)	0.08
	Fast	0.70(0.61/0.79)	0.06	0.74(0.64/0.85)	0.08
	Free scroll	–	–	0.66(0.61/0.70)	0.11
Horizontally	Slow	0.64(0.63/0.65)	0.15	0.69(0.70/0.69)	0.17
	Medium	0.66(0.66/0.65)	0.05	0.69(0.70/0.69)	0.08
	Fast	0.69(0.60/0.78)	0.14	0.76(0.69/0.84)	0.18
	Free scroll	–	–	0.69(0.68/0.71)	0.08

### Time efficiency

**Fig. 6:**
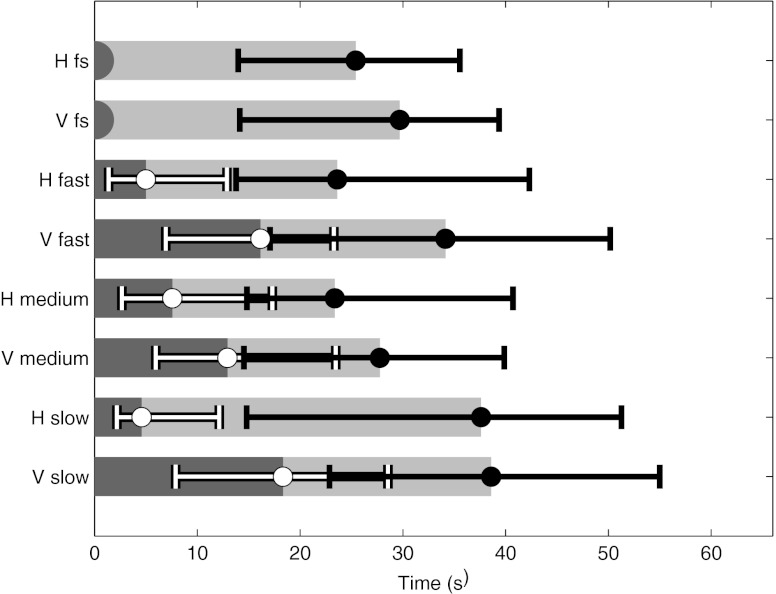
The median total analysis time (and quartiles) for all observers and conditions for all case types combined. The leftmost data (*in white*) is the time spent in the initial cine loop, whereas the rightmost data (*in black*) is the total analysis time

The median total analysis time and time spent in the cine loop are presented in Fig. 6. The time spent in the cine loop was remarkably short compared with when combined with FS. The relative differences in the geometrical means along with confidence intervals (CI_95_) are presented in Table 2. The total analysis time for horizontal FS (median time, 25 s) was statistically significantly shorter than vertical FS (median time, 30 s), though not statistically significantly different from the horizontal fast and medium frame rates and vertical medium frame rate as indicated by their overlapping CI_95_. The slow viewing procedures and the fast vertical presentation mode were statistically significantly longer. Considering the different cases (using pooled data from all conditions), the median time for the abnormal cases was 24 s, whereas it was statistically significantly longer (*P* value of < 0.001) for normal cases (median time, 44 s). There was no statistically significant difference (*P* value of 0.22) between the abnormalities.

**Table 2:** Summary of the statistical analysis of the total analysis time. All conditions were compared relative to the vertical free scroll browsing. Note that all data are log transformed and the estimates correspond to the ratio between the given condition and vertical free scroll browsing

Presentation mode	Viewing procedure	Estimate	*P* value	CI_95_
Horizontally	Free scroll	0.89	0.013	(0.81–0.98)
Horizontally	Fast	0.96	0.342	(0.87–1.05)
Horizontally	Medium	0.99	0.892	(0.90–1.09)
Vertically	Medium	1.04	0.404	(0.95–1.15)
Vertically	Fast	1.16	0.002	(1.06–1.27)
Horizontally	Slow	1.17	0.001	(1.07–1.28)
Vertically	Slow	1.31	<0.001	(1.19–1.438)

### Visual attention and search

**Fig. 7:**
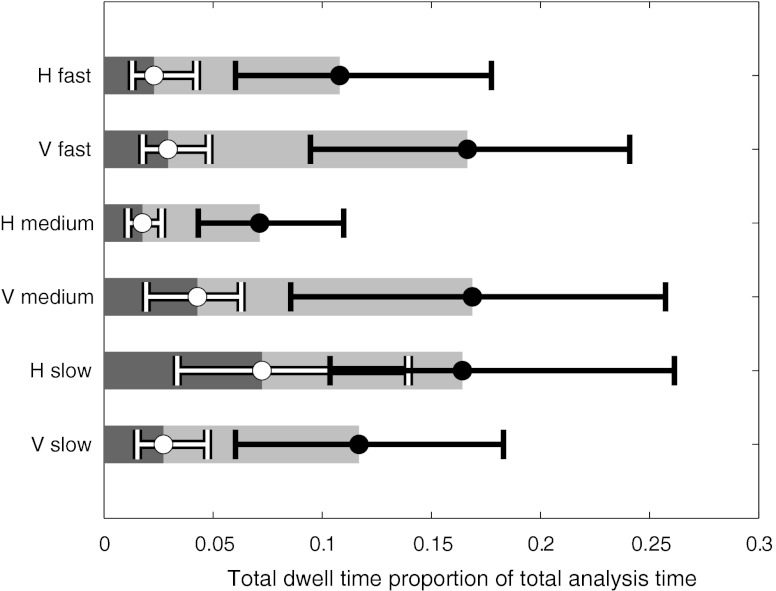
Normalised boxplots of the median total dwell time per viewing condition (excluding free scroll browsing) for the ROI (*leftmost*) and the c-ROI (*rightmost*)

As indicated in Fig. 7, median total dwell time in both the c-ROI and ROI normalised to the total analysis time was extremely short. A large spread of the data yielded no statistically significant differences for any condition. The normalised dwell time was longest for the vertical presentation with medium frame rate and shortest for the horizontal presentation at the same frame rate. However, no relevant conclusions could be drawn.

**Fig. 8:**
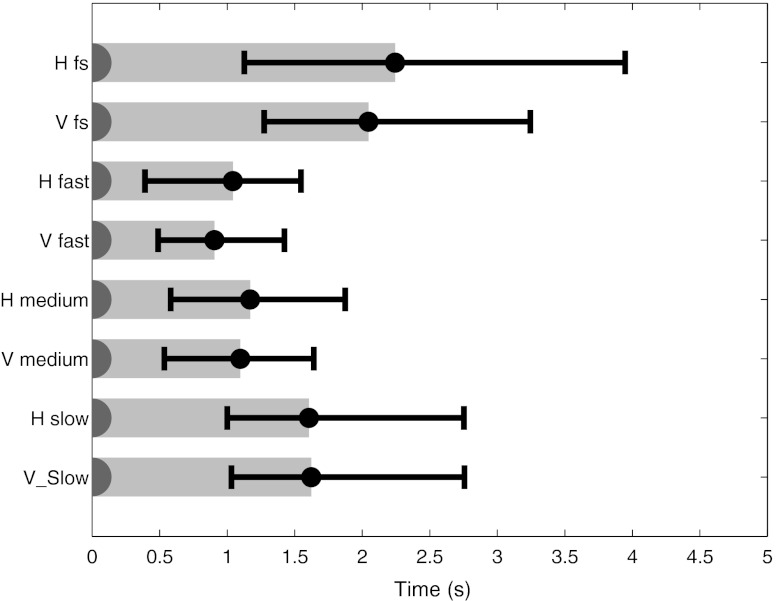
Median entry time (and associated quartiles) for all the conditions tested

The median entry time is presented in Fig. 8. The summary of the statistical analysis is presented in Table 3. In general, the entry time for all conditions was short. As hypothesised, higher frame rates relate to shorter entry times. The entry time during FS conditions (∼2 s) was statistically significantly longer compared with the fast and medium viewing conditions (∼1 s). The slow viewing procedures and FS in terms of entry time were statistically significantly longer. It took statistically significantly longer time (*P* value of 0.003) to allocate microcalcifications (median time, 1.55 s) compared with masses (median time, 1.12 s) using pooled data of all the conditions tested.

**Table 3:** Summary of statistical analysis of entry time. All conditions were compared relative to vertical free scroll browsing. Note that all data are log transformed and the estimates correspond to the ratio between the given condition and vertical free scroll browsing

Presentation mode	Viewing procedure	Estimate	*P* value	CI_95_
Horizontally	Fast	0.55	<0.001	(0.48–0.62)
Vertically	Fast	0.55	<0.001	(0.49–0.63)
Vertically	Medium	0.62	<0.001	(0.55–0.70)
Horizontally	Medium	0.64	<0.001	(0.56–0.72)
Vertically	Slow	0.80	<0.001	(0.71–0.89)
Horizontally	Slow	0.80	<0.001	(0.72–0.90)
Horizontally	Free scroll	1.08	0.134	(0.98–1.20)

**Fig. 9:**
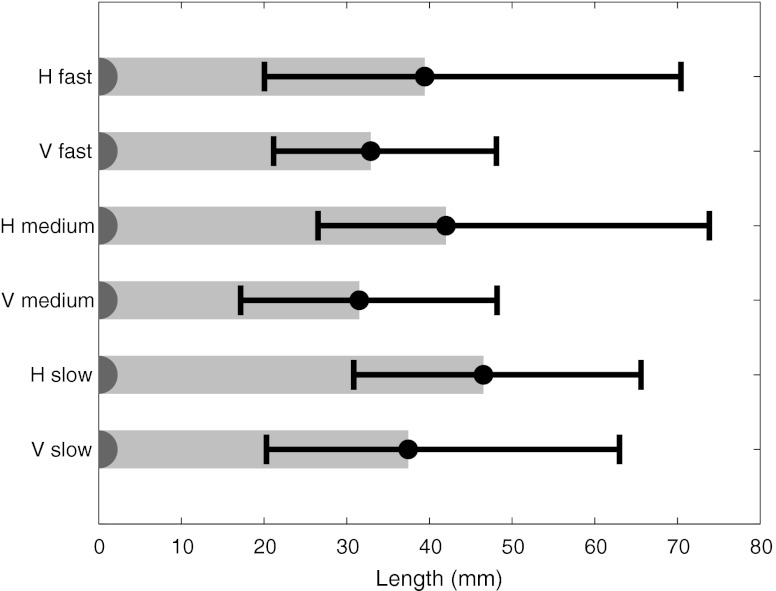
Median transition lengths (and associated quartiles) for all conditions (excluding free scroll browsing)

Regarding transition lengths, no statistical differences were observed between any conditions in the cine loop stage, probably because of a large spread of data. As observed by the median estimates in Fig. 9, the transition lengths in horizontal test conditions were somewhat longer, though not statistically significantly longer. The masses (median length, 42 mm) generated statistically significantly longer transitions (*P* value < 0.001) compared with microcalcification clusters (median length, 28 mm).

## Discussion

Studies on visual perception when reading radiology images are rather scarce [43]. Most of the studies have been related to mammography, and only a few of these studies have used eye tracking [22–26]. In this context our study can contribute to the implementation of BT in screening and improve our understanding of perception of 3D image volumes.

In summary, we observed no statistical differences in FOM as expected. When using FS only and FS combined with fast cine loop, our results suggest that horizontal viewing procedures reduce reading time compared with vertical viewing procedures. Furthermore, the results indicate that medium frame rates are comparable to fast ones in terms of reading time, whereas BT image volumes shown at slow frame rates are statistically significantly slower to read. Finally, faster cine loops lead to shorter entry times.

Our results showed that a horizontal presentation reduced the total analysis time, in particular for FS and for the fast cine loop speed. With a horizontal presentation mode, images are better aligned with the human visual field, which has wider extension horizontally than vertically [15, 16]. Peripheral vision can thus be used more efficiently to localise abnormalities in the BT image volumes, in particular where lesions appear and disappear abruptly in dynamic presentations. Such abrupt onsets are known to capture attention in a bottom–up manner [20, 44] and thus attract observers’ gazes. Therefore, it should be emphasised that the real benefit lies in viewing dynamic images rather than stationary images like 2D mammograms.

Cine loop may provide a better and faster global overview of possibly suspicious regions, and could speed up the process to select relevant suspicious regions during FS. Although if faster frame rates would be beneficial, we are still unaware when decisions are made. Our result indicated that FS alone or combined with a fast initial cine loop was more time efficient than other presentation modes. Surprisingly, little time was spent in the slow cine loop. Perhaps the observers felt uncomfortable awaiting appearances (and reappearances) of structures of interest or it did not provide a satisfying capture of visual attention. The relatively short time spent in the cine loop was also found in the other cine loop speeds, but this is possibly indicative of attracting visual attention more quickly. It took about twice the time for normal cases compared with abnormal cases, probably owing to lesion prevalence bias.

The constellation of observers may also be discussed. The amount of experience influences the general search task in radiological images. Nodine et al. [45] showed that experienced mammographers detected most breast lesions by global recognition within 25 s in mammograms, whereas less experienced observers took longer and that prolonged search increased the risk of error. Still, as BT is a new technique, even experienced breast radiologists have not yet received thorough experience in reading BT cases, especially not in reading BT image volumes horizontally. This was an experimental detection study and the observers only defined lesion presence.

Two lesion types (20 masses and 20 microcalcification clusters) were simulated, which leads to lower case variability. However, simulated lesions do not represent the wide range of different types of cancer growth patterns at BT [9], which is why our results have to be tested in a clinical patient population of cancers presented on BT. The observers were possibly biased because of the higher lesion prevalence than in a screening situation. In screening situations most of the cases are normal, and the total analysis time of these must be emphasised. Our intention is to proceed with a study based on the most promising reading conditions, involving experienced breast radiologists and real lesions.

Four viewing procedures and two presentation modes in BT image volumes have been evaluated. Our results indicate that viewing BT image volumes horizontally is an alternative to consider when utilising FS only or combined with a cine loop at fast frame rates. A fast cine loop also provided shorter entry times, detecting the lesions faster. The time spent in the cine loop was remarkably short. Considering time efficiency and measures of visual attention, the overall impression was that all conditions except for slow frame rates were found relevant.
